# The Royal Infirmary and Medical School, Newcastle-On-Tyne

**Published:** 1892-09-24

**Authors:** 


### THE INSTITUTIONAL WORKSHOP.

### WITHIN THE HOSPITALS.

### THE ROYAL INFIRMARY AND MEDICAL SCHOOL, NEWCASTLE-ON-TYNE.

[BY OUR SPECIAL COMMISSIONER.]

In the course of these reports it has been our practice to give a description of the institutions as we found them on the day of our inspection. The Newcastle Infirmary approaches the age of 150 years, which renders it undesirable and unnecessary to assume a similar course in regard to it. Those who have any hospital instruction will know that an old hospital constructed originally in 1755, and since added to from time to time, represents the "heap of buildings" type, and must necessarily contain features altogether out of date, and consequently beyond criticism.

It is satisfactory to know that last Monday the inhabitants of Newcastle assembled in public meeting to take into consideration what steps it was desirable to adopt with a view to securing for the poor of the town and neighbourhood a new hospital replete with every modern appliance and requirement. It must not, however, be supposed that old as parts of the Newcastle Infirmary really are that it does not possess good points. The most recent additions consist of (1) a block now devoted to the treatment of medical cases, which, like Guy's, the London, St. Bartholomew's, and other hospitals, contains wards on the back to back principle; and (2) a so-called temporary wing, consisting of a large pavilion of one storey divided down the centre by a dwarf partition, forming two large wards under one roof on the same plan as those in the first-mentioned block. The floors of the temporary (?) pavilion, which, like that of most erections of the kind, has been kept constantly in use for a number of years, and is at present in a good state of preservation, and leaves absolutely nothing to be desired. The atmosphere of the ward, its general appearance, and hygienic conditions are satisfactory; and although the lavatories, baths, and fittings are out of date and unsuitable to a hospital in our opinion, still this temporary pavilion has many good points which deserve recognition. In the same way the block of medical wards in the wing referred to are excellently proportioned, well lighted, with an abundance of superfical area and cubic space, and apart from certain defects, which might be readily remedied, they are on the whole satisfactory as wards of this type. The defects embrace, of course, the absence of cross ventilation, which could be greatly modified, if not entirely remedied, by artificial means; and the situation of the laundry and out-patients department or of portions of one or both in the basement of the buildings in which the wards are situated.

Now we cannot, of course, foresee what decision the inhabitants of Newcastle may come to on the vexed question of an alternative site which has arisen in Newcastle as it has done at Manchester. Holding the opinion from a large and varied experience, that it is desirable to curtail rather than to extend building operations, even in cases where old hospitals have to be reconstructed, we feel that the people of Newcastle, seeing that hospitals are intended to meet to the utmost the requirements of the poor, would be well advised to think once, twice, nay thrice, before they permit the Royal Infirmary to be removed from its present site. The retention of the old site will secure an abundance of space surrounding the new infirmary, and would enable the old block referred to to be retained, and the after reconstruction to be occupied by a certain class of medical cases which must be admitted to the infirmary, and which would do as well in these wards under the altered circumstances as they could be reasonably expected to do in the most modern pavilion which science can devise. Further than that, the demolition of the other parts of the existing infirmary, the taking up of the whole of the present drains, which are no doubt mainly brick culverts, the removal of several feet of the soil and its replacement by fresh earth, which should then be covered with concrete, would make the site an admirable one for the new infirmary buildings. If, as a correspondent to the local papers declares, patients are sent out at present from the infirmary in a feeble condition of health, that may be due altogether to the great pressure existing upon the available beds; and if it is due to other considerations, then the precautions we have suggested for the erection of new buildings on the present site, with the addition, possibly, of convalescent accommodation within easy reach of Newcastle, would meet this particular objection. To remove altogether from the present site, which is held under a lease from the Corporation, would entail enormous additional expenditure, in our opinion altogether out of proportion to the advantages likely to result from a deviation from this course. We shall therefore hope to see that a representative committee is appointed to confer with the medical staff and to bring up a report to an adjourned meeting. This will afford an opportunity for the whole of the special circumstances to be adequately dealt with, and we make no doubt that the ultimate decision will be wise and at the same time worthy of the best traditions of Newcastle.

#### SPECIAL FEATURES OF THE PRESENT WORK.

No one can visit Newcastle Infirmary, and, after a careful inquiry into the results of the treatment, a close observation of the medical and surgical practice, and a thorough inspection of the conditions of the buildings and the accommodation they contain, without being struck with the conviction that nowadays the science of medicine has so developed that successful medical and surgical treatment is largely independent of any mere question of buildings. It is remarkable and satisfactory that any advanced student who wanted to make himself practically acquainted with modern methods of treatment and their wise application, could not do better than take out a six months' course at the Newcastle Infirmary. The organisation of the surgical practice is most efficient, and the severer operations can here be done under most favourable circumstances, because the members of the staff co-operate to make them a success, and the most eminent surgeons could not fail to congratulate these gentlemen on the high state of efficiency to which they have attained. We were present for some hours in the operating theatre, and were much struck with the excellence which characterised everything connected with the work there carried on. It is not too much to say that a patient in the Newcastle Infirmary, however severe may be his disease, will receive at the hands of the surgical staff the maximum of aid, and that here a patient has as good a chance of recovery as he could secure anywhere in the world.

Then again, we could wish that some of those critics who have expected so much by elaborating a paper system of efficiency for hospital nurses could be brought to see the disadvantageous conditions under which the nursing staff of this Infirmary have carried on their work for years, and at the same time be made to realise that these devoted women have done their duty nobly without complaint and with a maximum of efficiency. The Committee are fully alive to the structural defects and difficulties which present circumstances entail upon them. They have cheerfully done their best to accommodate the staff, but when all has been done that could be done the conditions compared with those offered by the best London hospitals leave much to be desired. Despite this, as we have said, the nursing appears to be excellent, and no member of the staff has ever even thought of complaining of the hardness of their lot, because they have felt that their first duty was the patients, that they had every confidence in the Committee, and that so soon as it was possible the work would be carried on under improved conditions in the new buildings, which are so urgently needed. In these circumstances it is not surprising to find that the rate of mortality from operations at the Newcastle Infirmary is remarkably low, and although cases of pneumonia have done very badly of late years, that cannot properly be made an argument against the retention of the wing which we have specially referred to, seeing that provision can be made for the severer cases in special wards of the new buildings about to be erected.

Medical School.

Mr. Page, who is the Registrar of the Medical School, has taken a very great interest in the new buildings which have been recently erected for the accommodation of the students. These buildings, of which we propose to give a plan and full particulars in a subsequent issue, are replete with every modern appliance. Attached is an excellent dissecting-room, for example—one of few in the country —and altogether we are convinced that it is desirable in the best interests of students to direct their attention, and that of their friends, to the Newcastle Medical School. It is in connection with the University of Durham, a circumstance of no little importance in itself, as it enables the students to take a good degree, and seeing the eminence and capacity of the present staff of clinical teachers and professors, we are confident that the Newcastle General Medical School deserves to secure a much larger entry than has yet fallen to its lot. Such a result is likely to be hastened by the erection of a new hospital.

